# Real world evidence of Lenvatinib + anti PD-1 as an advanced line for metastatic melanoma

**DOI:** 10.3389/fonc.2023.1180988

**Published:** 2023-05-18

**Authors:** Ronen Stoff, Nethanel Asher, Shachar Laks, Yael Steinberg, Jacob Schachter, Ronnie Shapira-Frommer, Shirly Grynberg, Guy Ben-Betzalel

**Affiliations:** Sheba Medical Center, Ella Lemelbaum Institute of Immuno-Oncology, Ramat Gan, Israel

**Keywords:** melanoma, Lenvatinib, immunotherapy, anti PD1, pembrolizumab

## Abstract

**Introduction:**

Immunotherapy has revolutionized the prognosis of patients with metastatic melanoma. To date, the most active regimen is the combination of ipilimumab + nivolumab (ipi-nivo) achieving a response rate of nearly 60% and a median survival (OS) of 6 years. However, approximately 40% of patients experience primary resistance, while around 50% experience secondary resistance, highlighting the need for an effective second-line treatment option The recently published results on the use of lenvatinib + pembrolizumab in the advanced line setting led to the adoption of this regimen at our institution. Here we present our experience with this regimen, focusing on efficacy and safety.

**Methods:**

Electronic medical records of patients treated at a tertiary referral melanoma center, with at least one cycle of anti PD-1 + lenvatinib from 2020 to 2023 were analyzed for baseline demographic characteristics, disease related characteristics and treatment outcomes.

**Results:**

Forty-two patients were identified. The Response rate (RR) was 28% and the disease control rate was 38%. Responses were seen across different melanoma subtypes, including 67% in acral melanoma, 20% in uveal melanoma, and 25% in mucosal melanoma. Patients with a more aggressive disease manifested by elevated lactate dehydrogenase (LDH) achieved a RR of 26%, while patients with active central nervous system (CNS) metastases had a RR of 31%, and an intra-cranial RR of 23%. Responses were seen across lines of treatment, with a 25% RR in the second and third lines, and a 36% RR in the fourth and fifth lines. The median progression free survival was 3 months, and the median survival was 11 months. The treatment was not easily tolerated with 31% of the patients experiencing grade 3-4 toxicity, which was manageable through dose interruptions and reductions. Only 7% of patients discontinued the treatment due to toxicity.

**Conclusion:**

Lenvatinib in combination with anti-PD1 had demonstrated both relative safety and efficacy in patients with metastatic melanoma of all subtypes in the advanced line setting. We are eagerly anticipating the mature results of the LEAP-004 study hoping that this regimen will receive regulatory approval, paving the way for its widespread adoption in daily practice worldwide.

## Introduction

Malignant melanoma is the 5^th^ most common cancer diagnosed in the US annually (1), and for many years metastatic melanoma has been considered an aggressive and fatal malignancy for which no durable effective treatment was available. The overall median 1- and 5-year survival rates were 41% and 22% respectively as late as 2011.

The introduction of novel immunotherapy agents in the last decade has revolutionized the treatment of melanoma with a marked increase in patient overall survival (2). The first agent to be introduced was ipilimumab, an anti Cytotoxic T-lymphocyte associated protein 4 (CTLA-4) agent which has shown improved survival in 2010, albeit with a significant toxicity profile and low response rates (3). The development of anti programmed death-1 (PD-1) agents such as nivolumab and pembrolizumab have further increased the efficacy and exhibit a safer toxicity profile (4, 5).

The combination of nivolumab and ipilimumab (ipi-nivo) was tested in the pivotal Checkmate 067 trial and demonstrated the most promising outcome to date, with an enhanced overall response rate (ORR) of 58% and a 7.5 year overall survival (OS) rate of 48% (6, 7). Despite these encouraging long-term results, 36% of patients do not respond to the combination with 12% achieving Stable disease (SD) as best response and 24% having progressive disease (PD) as best response (6).

While for those patients who are resistant to first line single agent anti PD-1, second line immunotherapy based on anti-CTLA4 agents (alone or in combination) could serve as an adequate salvage therapy with response rates up to 20-30% (8–11), treatment options for patients who exhibit primary resistance to one of the combination immunotherapy regimens are limited. Randomized trials, as well as some retrospective studies looking at the sequential use of different types of immunotherapy show a limited efficacy in the 2^nd^ line setting (12, 13).

For patients with an activating BRAF mutation, targeted therapy with a combination of BRAF-MEK inhibitors is the best 2^nd^ line option, with two studies evaluating the best sequence demonstrating superior results for 1^st^ line immunotherapy followed by 2^nd^ line targeted therapy (14, 15). Yet, the problem of treatment resistance is very common for those patients treated with targeted therapy with a median PFS of all available BRAF-MEK inhibitor combinations of about 12 months (16–18).

Different approaches have been tried in clinical trials as an alternative 2^nd^ or 3^rd^ line options for those patients whose disease has progressed on immunotherapy (19–21), including the use of adoptive cell therapy (ACT) with Tumor Infiltrating Lymphocytes (TIL) which had shown superior outcomes compared to ipilimumab (22). One such recent study that has shown promising results is the Phase II LEAP-004 study which evaluated the combination of the anti-PD1 agent pembrolizumab with the multi-targeted tyrosine kinase inhibitor (TKI) Lenvatinib. Lenvatinib targets several cancer-associated pathways, including the vascular endothelial growth factor receptor (VEGFR) and the fibroblast growth factor receptor (FGFR) (23). In 2013 Lenvatinib has shown a low ORR of 9% when given as a single agent for previously treated metastatic melanoma patients (24) and it’s use as a single agent in melanoma was not pursued. In 2019 a much more promising RR of 48% was achieved when combined with pembrolizumab in the melanoma arm of a phase Ib/II study (25). The improved results of the combination treatment were attributed to the VEGFR and FGFR blockade, which shifts the tumor microenvironment to an immune-stimulatory state, thus improving the efficacy of PD-1 blockade. This rational was also demonstrated in several pre-clinical mouse model studies (26–29). The promising results of the combination have prompted the initiation of numerous trials across different cancer types, with encouraging results already published in renal cell carcinoma, hepatocellular carcinoma and endometrial carcinoma (30–32). These results served as the basis for the Phase II LEAP-004 study.

The LEAP-004 study enrolled 103 previously treated metastatic melanoma patients. All patients were previously treated with an anti PD-1 agent, either alone or in combination with the anti-CTLA4 agent ipilimumab. Forty-two percent of patients have received only 1 prior line and the other 58% had 2 or more previous lines. For the entire cohort the ORR was 21% and a higher ORR of 33% was shown in patients previously treated with the ipi-nivo combination (32% of patients). For the entire cohort the median PFS was 4.2 months, and the median OS was 14 months. Grade 3-5 treatment related adverse events were seen in 45% of patients with the most common one being hypertension in 21% of patients (33). Following the release of the first interim analysis in 2020 (34) our institution began using the pembrolizumab-lenvatinib combination for patients who have exhausted all other options. The use of this regimen was approved by the local ethics committee. In addition, exemption was granted to use nivolumab as a substitute for pembrolizumab in cases where patients were unable to receive reimbursement for pembrolizumab from their insurer or had to fund their therapy (because nivolumab is less expensive and more readily available in Israel).

The objective of this study is to describe our real-world outcomes obtained though the implementation of lenvatinib and anti PD-1 as an advanced-line treatment for metastatic melanoma.

## Materials and methods

Electronic medical records of metastatic melanoma patients treated at the Ella Lemelbaum Institute for immuno-oncology with the combination of Lenvatinib and one of the anti PD-1 agents: nivolumab or pembrolizmuab from the year 2020 onward were collected. Records were analyzed for baseline parameters including demographics, Melanoma related data (e.g. disease stage, histological subtype) and all data regarding treatment with the Lenvatinib combination (including timing of treatment, dosage and dose reductions and side effects). Treatment response was described by the treating physician with either clinical response (for palpable disease) or radiological response per the immune response evaluation criteria - iRECIST v1.1 (35). Intra-cranial response was evaluated using brain magnetic resonance imaging (MRI). All subsequent visits were screened for follow up results including subsequent lines and survival. Data cut-off was January 23^rd^, 2023.

Progression-free survival (PFS) and overall survival (OS) curves were assessed using the Kaplan-Meier method. Toxicity grading was done using the common terminology criteria for adverse events (CTCAE) v.5 (36).

All statistical analyses were done with MedCalc Version 20.218.

Data was collected and analyzed in accordance with the local IRB approval (SMC-4387-17).

## Results

Forty-two patients that received at least one cycle of anti PD-1 combined with Lenvatinib were identified and their medical records were analyzed. All patients were treated outside of a clinical trial setting and had access to Lenvatinib *via* their private health insurance (if available) or out-of-pocket expenses. Baseline demographic and disease related parameters are described in **Table 1**.

**Table 1:** Baseline characteristics.

	n=42 (100%)
Age, years (Median)	66 (28-80)
Melanoma subtype n (%) Cutaneous Acral Mucosal Uveal Unknown	26 (62%) 6 (14%) 4 (10%) 5 (12%) 1 (2%)
BRAF V600 Mutation n (%) V600E/K Mutated Non V600 Mutated Wildtype	13 (31%) 1 (2%) 28 (67%)
ECOG PS n (%) 0 1 2 3	21 (50%) 12 (29%) 8 (19%) 1 (2%)
Serum LDH n (%) Normal range Elevated <X2 UNL Elevated >X2 UNL Unknown	14 (33%) 19 (45%) 8 (19%) 1 (2%)
Metastatic at presentation n (%) Yes No	7 (17%) 35 (83%)
Active CNS metastases n (%) Yes No	13 (31%) 29 (69%)
Liver metastases n (%) Yes No	17 (40%) 25 (60%)
Bone metastases n (%) Yes No	9 (21%) 33 (79%)
Lenvatinib + Anti PD-1 line n (%) 2^nd^ 3^rd^ 4^th^ 5^th^	15 (35%) 13 (31%) 7 (17%) 7 (17%)
Previous lines of treatment Ipilimumab+Nivolumab combination Single agent anti PD-1	37 (88%) 5 (12%)

PS, performance status; LDH, lactate dehydrogenase; UNL, upper normal limit; CNS, central nervous system.

The median age was 66 (28-80). Twenty-two patients were male (52%). Melanoma subtypes were cutaneous (62%), acral (14%), uveal (12%), mucosal (10) and unknown primary melanoma (2%). 13 patients (31%) had a BRAF V600 mutation (12 had V600E and 1 had V600K), 1 patient (2%) had a BRAF G469V mutation and the other 28 patients had wild-type (WT) BRAF (67%). At treatment initiation 13 patients (31%) had active CNS metastases, for whom the response was evaluated both systemically and intra-cranially (using brain imaging before and after treatment initiation). 17 patients (40%) had liver metastases and 9 patients (21%) had bone metastases with 8 patients (19%) having both. The ECOG Performance status (PS) was 0-1 in 33 patients (79%) and 2-3 in the other 9 patients (21%). Baseline levels of Lactate Dehydrogenase (LDH) at treatment initiation were within normal range for 33% of the patients, while 64% had elevated LDH (45% elevated up to twice the upper normal limit and 19% elevated more than twice the upper normal limit).

37 patients (88%) were previously treated with the ipi-nivo combination (17 as first line, 12 as second line, 5 as third line and 3 as fourth line). The remaining 5 patients (12%) were all treated with single agent anti PD-1 in the first line setting, with two of them receiving ipilimumab single agent as the second line.

The median line of treatment with Lenvatinib + anti PD-1 was 3 (2–5). The anti PD-1 agent used was Pembrolizumab for 26 patients (62%) and Nivolumab for the other 16 patients (38%). The median follow-up time was 6 months (1-25).

Response assessment was done using imaging for 39 patients (93%), while the other 3 patients were assessed clinically. All three had shown rapid clinical disease progression and were deemed as PD prior to their first scheduled imaging. A total of 6 patients received only 1 or 2 cycles of treatment before treatment was discontinued due to PD (clinical or radiographic).

The best overall response rate was 28% (21% PR, 7% CR) and another 10% achieved SD, leading to a total of 38% disease control rate (DCR). The median duration of response was 5.5 months (range 2-21), with 5 patients still ongoing at data cut-off.

Response patterns according to subgroups are described in **Table 2**.

**Table 2:** Response patterns according to subgroups.

	n (% of all patients)	ORR n (%)
Age >65 <65	23 (55%) 19 (45%)	8 (35%) 4 (21%)
Melanoma subtype n (%) Cutaneous Acral Mucosal Uveal Unknown	26 (62%) 6 (14%) 4 (10%) 5 (12%) 1 (2%)	6 (23%) 4 (67%) 1 (25%) 1 (20%) 0 (0%)
BRAF V600 Mutation n (%) V600E/K Mutated Non V600 Mutated Wild type	13 (31%) 1 (2%) 28 (67%)	1 (8%) 1 (100%) 10 (36%)
ECOG PS n (%) 0 1 2 3	21 (50%) 12 (29%) 8 (19%) 1 (2%)	7 (33%) 3 (25%) 2 (25%) 0 (0%)
Serum LDH n (%) Normal range Elevated <X2 UNL Elevated >X2 UNL Unknown	14 (33%) 19 (45%) 8 (19%) 1 (2%)	5 (36%) 5 (26%) 2 (25%) 0 (0%)
Metastatic at presentation n (%) Yes No	7 (17%) 35 (83%)	2 (29%) 10 (29%)
Active CNS metastases n (%) Yes No	13 (31%) 29 (69%)	4 (31%) 8 (28%)
Liver metastases n (%) Yes No	17 (40%) 25 (60%)	4 (23%) 8 (32%)
Bone metastases n (%) Yes No	9 (21%) 33 (79%)	1 (11%) 11 (33%)
Lenvatinib + Anti PD-1 line n (%) 2^nd^ 3^rd^ 4^th^ 5^th^	15 (35%) 13 (31%) 7 (17%) 7 (17%)	3 (20%) 4 (31%) 3 (43%) 2 (29%)
Anti PD-1 agent used n (%) Pembrolizumab Nivolumab	26 (62%) 16 (38%)	8 (31%) 4 (25%)
Previous line n (%) Ipilimumab-Nivolumab Single agent Anti PD-1	37 (88%) 5 (12%)	9 (24%) 3 (60%)
Type of immunotherapy resistance n (%) Primary Acquired	22 (52%) 20 (48%)	8 (36%) 4 (20%)
Grade 3-4 toxicity n (%) Yes No	13 (31%) 29 (69%)	6 (46%) 6 (21%)

PS, performance status; LDH, lactate dehydrogenase; UNL, upper normal limit; CNS, central nervous system.

Responses according to the metastatic site were variable with 4 out of 17 patients with liver metastases showing a response (23%) and only 1 out of the 9 patients with bone metastases showing a response (11%). There were 8 patients who had both liver and bone metastases, of which only 1 showed a response (12.5%).

Patients with active CNS metastases (n=13) were all treated with stereotactic radiosurgery (SRS) for all active or growing CNS lesions at the same period as initiating the systemic therapy. (plus or minus 2 weeks). None of these patients undergo a neurosurgical intervention. These patients had a systemic response rate of 31% and an intra-cranial response rate of 23% (yet all were simultaneously treated with SRS as mentioned).

Responses according to melanoma subtype also varied with a 23% for cutaneous melanoma, a 67% response rate for acral melanoma, 25% for mucosal melanoma and 20% for uveal melanoma.

**Figure 1:**
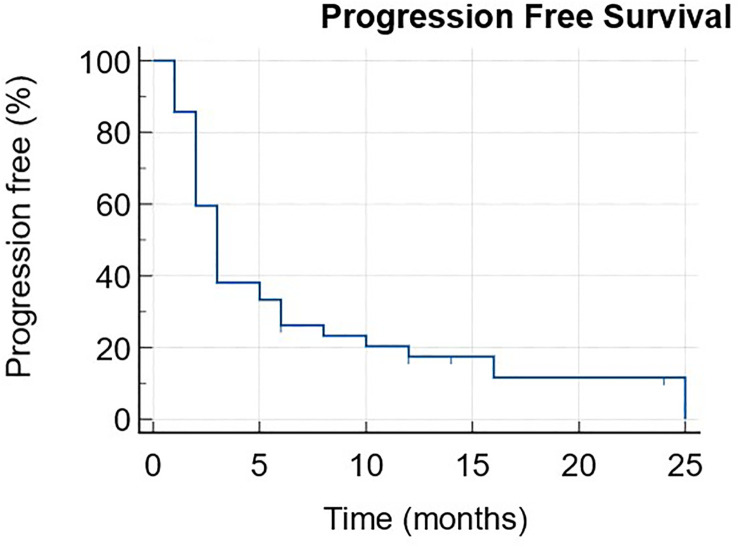
Progression free survival.

**Figure 2:**
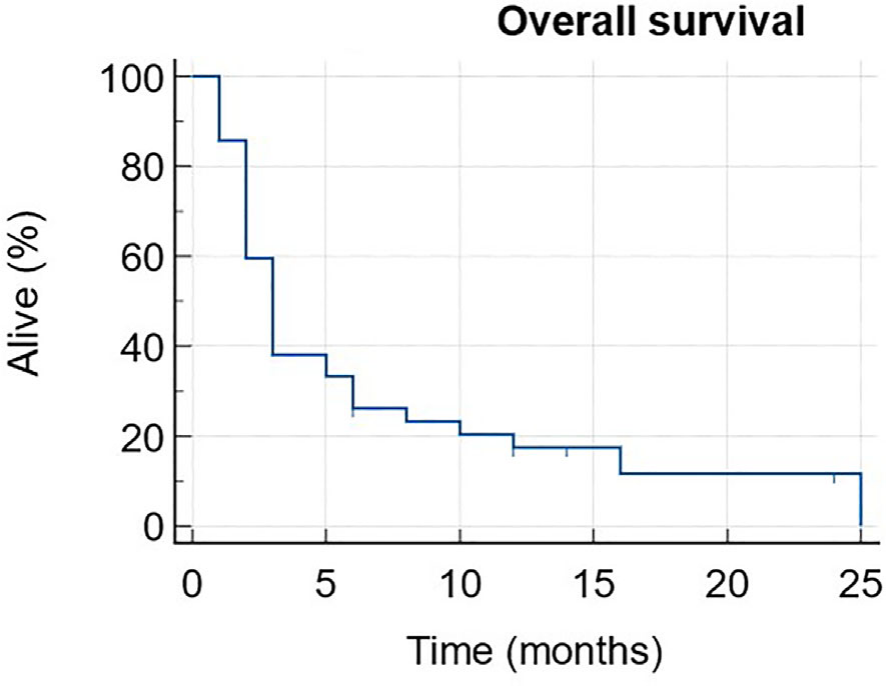
Overall survival.

The median PFS was 3 months (1-25) with 6 patients still ongoing at data cut-off (**Figure 1**). The median OS was 11 months (1-NR) with 11 patients still alive at data cut-off (**Figure 2**).

The median dose of Lenvatinib used was 10 mg (8–20), with 14 patients (33%) staying at the starting dose of 20 mg with no dose interruptions and the other 28 (67%) having dose reductions. The most common cause for dose reduction was toxicity in 19 patients (45%) with 3 of them stopping treatment due to toxicity (one patient experienced perforation requiring emergency surgery, one developing grade 4 skin toxicity and one who stopped both agents due to severe neurological toxicity and continued only Lenvatinib with a reduced dose of 10 mg). Nine patients (21%) received a reduced dose of 10 mg due to financial difficulties obtaining the full dose as the treatment is not reimbursed in Israel by the ministry of health. Toxicity of all grades was seen in 26 patients (62%) with 13 patients (31%) experiencing Grade 3-4 toxicity. The most common G3-4 side effects seen were proteinuria and hypertension (3 patients each – 7%), fatigue, skin reaction and diarrhea (2 patients each – 5%). One patient experienced a life-threatening colonic perforation and another patient suffered two episodes of cerebrovascular attacks (CVA). The most common treatment related adverse events are described in **Table 3**.

**Table 3:** Treatment related adverse events.

Toxicity	All grades n (%)	G3-4 n (%)
Fatigue	9 (21%)	2 (5%)
Skin reactions (including HFS)	6 (14%)	2 (5%)
Diarrhea	6 (14%)	1 (2%)
Hypertension	5 (12%)	2 (5%)
Proteinuria	4 (10%)	3 (7%)
Rheumatologic toxicity	3 (7%)	1 (2%)
Elevated LFTs	2 (5%)	0 (0%)
Anemia	1 (2%)	1 (2%)
Perforation	1 (2%)	1 (2%)

G3-4, Grade 3-4; LFTs, liver function tests; HFS, hand & foot syndrome.

## Discussion

The rapid incorporation of immunotherapy for malignant melanoma has changed the oncological outcomes drastically with improved PFS and OS for a major portion of patients. Nevertheless, a significant portion of the patients derive little if any benefit from these novel agents and require better treatment alternatives. Lenvatinib is a multi-targeted TKI that inhibits VEGF and FGFR among other targets. While attempts to use Lenvatinib as a single agent for metastatic melanoma have shown minimal efficacy, the combination with anti PD-1 seems to be more promising with current phase II available data showing a 21% ORR in the second- or third-line setting. We collected and analyzed our experience using Lenvatinib in combination with anti PD-1 agents in the real-world setting with a very diverse group of patients. The ORR was 28% which is even more impressive taking into consideration the fact that our population consisted of all different melanoma subtypes, almost all (88%) pre-exposed to the Ipilimumab-Nivolumab combination. Of note is the fact the responses were seen across all subtypes with a 67% RR for acral melanoma, 23% RR for cutaneous melanoma and 20-25% RR for uveal and mucosal melanomas (albeit with a very small number of patients). Responses were also seen in heavily pretreated patients, with a 36% RR in the 4^th^ and 5^th^ lines and a 25% RR in the 2^nd^ and 3^rd^ line. Even patients with a more aggressive disease characterized by elevated LDH seem to benefit from this combination regimen with a 26% RR, as well as those with active CNS metastases for whom a 31% response rate was seen systemically with an intra-cranial response rate of 23% (with one patient achieving only systemic response that was treated with SRS for the CNS progression while continuing treatment with the regimen). All CNS patients received SRS to all active brain metastases at the same time of treatment initiation with lenvatinib, which prevents a true assessment of the intra-cranial efficacy of this regimen by itself. Patients with liver metastases, a known poor predictive factor for response to immunotherapy responded roughly the same as those with no liver metastases (23% vs. 32%), hinting that the use of lenvatinib might positively affect the immune response in the immunosuppressive tumor microenvironment in the liver. Patients with a BRAF V600 mutation seem to derive a much more modest benefit as only 1 out of the 13 patients with a V600 mutation responded to the treatment (8%), in contrary to those with BRAF WT who had a 36% RR. Whether this is coincidental or related to cross-pathway resistance mechanism needs to be further elucidated. Response rates were about the same when using either Pembrolizumab (31%) or Nivolumab (25%), reaffirming the notion that the two drugs may by interchangeable.

As previously reported the treatment is quite toxic with 31% of the patients developing G3-4 toxicity, a lower percentage than previously reported, which might be attributed to the fact that about 21% of the patients received a reduced dose of 10 mg due to financial difficulties obtaining Lenvatinib. Most toxicities were manageable with dose interruptions and reductions, yet it is worth mentioning the one patient who suffered two CVAs while on treatment. Only 3 patients (7%) had to stop Lenvatinib due to toxicity. It is worth mentioning a numerical higher response rate was seen in those patients who developed G3-4 side effects (46% vs. 21%), yet the explanation for this difference is unclear and might be related to the known correlation between immune related adverse events and immunotherapy efficacy (37, 38).

## Conclusions

Our single center series of 42 patients treated with anti PD-1 + Lenvatinib shows promising results, yet they should be interpreted within the built-in caveats in the study design. It is a retrospective study including a small cohort of patients, limiting its utility in evaluating the efficacy of this regimen across different subgroups and melanoma subtypes. The results were also influenced by the fact that this regimen is not reimbursed by the public health system in Israel, leading to a selection bias of patients who could either afford the cost of the treatment or own a second non-public health insurance. This has also affected the dose of Lenvatinib taken by a portion of the patients who had to pay out of pocket for the medicine, usually leading to a financial dose reduction.

Nevertheless, the diverse group of patients represented in this cohort shows a more inclusive real-world line-up than pharma based clinical trials, emphasizing this regimen’s potential in advanced line and possibly 1^st^ line metastatic melanoma patients.

This regimen seems safe (with proper dose modifications) and efficient in the advanced line setting for metastatic melanoma patients of all subtypes and the mature results of the LEAP-004 study are eagerly awaited with a hope for regulatory approval for this regimen soon, leading to its incorporation in the daily practice worldwide.

## Data availability statement

The datasets presented in this article are not readily available because The data cannot be shared due to Israeli local legislative restrictions on patient data sharing. Requests to access the datasets should be directed to Ronen Stoff, Ronen.stoff@sheba.gov.il.

## Ethics statement

The studies involving human participants were reviewed and approved by Sheba medical center IEC, approval number: SMC-4387-17. Written informed consent for participation was not required for this study in accordance with the national legislation and the institutional requirements.

## Author contributions

RS - Authorship, Data analysis, Statistical planning, Manuscript preparation, Draft revision. NA - Database management, Manuscript preparation. SL - Manuscript preparation, Draft revision. YS - Manuscript preparation, Draft revision. JS - Manuscript preparation, Draft revision. RS-F - Statistical analysis, Draft revision. SG - Statistical planning, Manuscript preparation, Draft revision. GB-B - Statistical planning, Manuscript preparation, Draft revision. All authors contributed to the article and approved the submitted version.
